# The effect of standardized food intake on the association between BMI and ^1^H-NMR metabolites

**DOI:** 10.1038/srep38980

**Published:** 2016-12-14

**Authors:** Bianca A. M. Schutte, Erik B. van den Akker, Joris Deelen, Ondine van de Rest, Diana van Heemst, Edith J. M. Feskens, Marian Beekman, P. Eline Slagboom

**Affiliations:** 1 Department of Molecular Epidemiology, Leiden University Medical Center, 2300 RC Leiden, The Netherlands; 2 The Delft Bioinformatics Lab, Delft University of Technology, 2628 CD Delft, The Netherlands; 3 Max Planck Institute for Biology of Ageing, Köln, Germany; 4 Division of Human Nutrition, Wageningen University, 6700 EV Wageningen, The Netherlands; 5 Department of Internal Medicine, section Gerontology and Geriatrics, Leiden University Medical Center, 2300 RC Leiden, The Netherlands

## Abstract

Multiple studies have shown that levels of ^1^H-NMR metabolites are associated with disease and risk factors of disease such as BMI. While most previous investigations have been performed in fasting samples, meta-analysis often includes both cohorts with fasting and non-fasting blood samples. In the present study comprising 153 participants (mean age 63 years; mean BMI 27 kg/m^2^) we analyzed the effect of a standardized liquid meal (SLM) on metabolite levels and how the SLM influenced the association between metabolites and BMI. We observed that many metabolites, including glycolysis related metabolites, multiple amino acids, LDL diameter, VLDL and HDL lipid concentration changed within 35 minutes after a standardized liquid meal (SLM), similarly for all individuals. Remarkable, however, is that the correlations of metabolite levels with BMI remained highly similar before and after the SLM. Hence, as exemplified with the disease risk factor BMI, our results suggest that the applicability of ^1^H-NMR metabolites as disease biomarkers depends on the standardization of the fasting status rather than on the fasting status itself. Future studies are required to investigate the dependency of metabolite biomarkers for other disease risk factors on the fasting status.

Multiple studies have shown that levels of metabolites can predict the risk of diseases such as diabetes^1^,^2^,^3^ and cardiovascular disease^4^, and associate with disease risk factors including insulin resistance^5^ and body mass index (BMI)^6^. In a study comprising 12,664 adolescents and young adults (16–39 years old, 51% women), Würtz *et al*.^6^ found an association between BMI and levels of cholesterol, lipids, fatty acids, ketone bodies, glycolysis related metabolites and amino acids. Hence, metabolite levels seem to reflect information about one’s metabolic state and health.

Generally, fasting blood samples are studied for associations with metabolite levels. However, some clinical studies and longitudinal cohorts have non-fasting blood samples, which raises the issue whether there is a systematic change in levels due to a meal or that the underlying correlation structure of metabolite levels with investigated phenotypes also changes^1^. It would be valuable using both studies with fasted and non-fasted samples in meta-analyses of the association between metabolites and disease (risk factors). Previously, some studies have investigated the effect of different meals on metabolite levels^7^,^8^,^9^,^10^. However, the intra-individual effects of standardized food intake on the association between metabolites and a disease risk factor have not yet been tested.

Therefore, in this study, we measured metabolites in a group of 153 individuals participating in the Growing Old Together Study (GOTO) at fasting status and at 35 minutes after a standardized liquid meal (SLM). First, we investigated how ^1^H-NMR metabolite levels change after a SLM. Second, we determined the association between metabolite levels and BMI, as an example of a disease risk factor, in both fasting and postprandial status. Third, we compared the effect sizes of the association with BMI of each metabolite between the fasting and postprandial status.

## Results

We have analyzed data of 153 participants of the GOTO study of whom BMI and ^1^H-NMR metabolite levels before and after the SLM were available. The mean age of the participants was 63 years, mean BMI was 27 kg/m^2^ (SD 2.4) and 15 participants (10%) were obese (BMI>30 kg/m^2^) (Table 1). Mean blood pressure, cholesterol and glucose levels were in the normal range.

### Metabolite levels change after the standardized liquid meal

**Figure 1:**
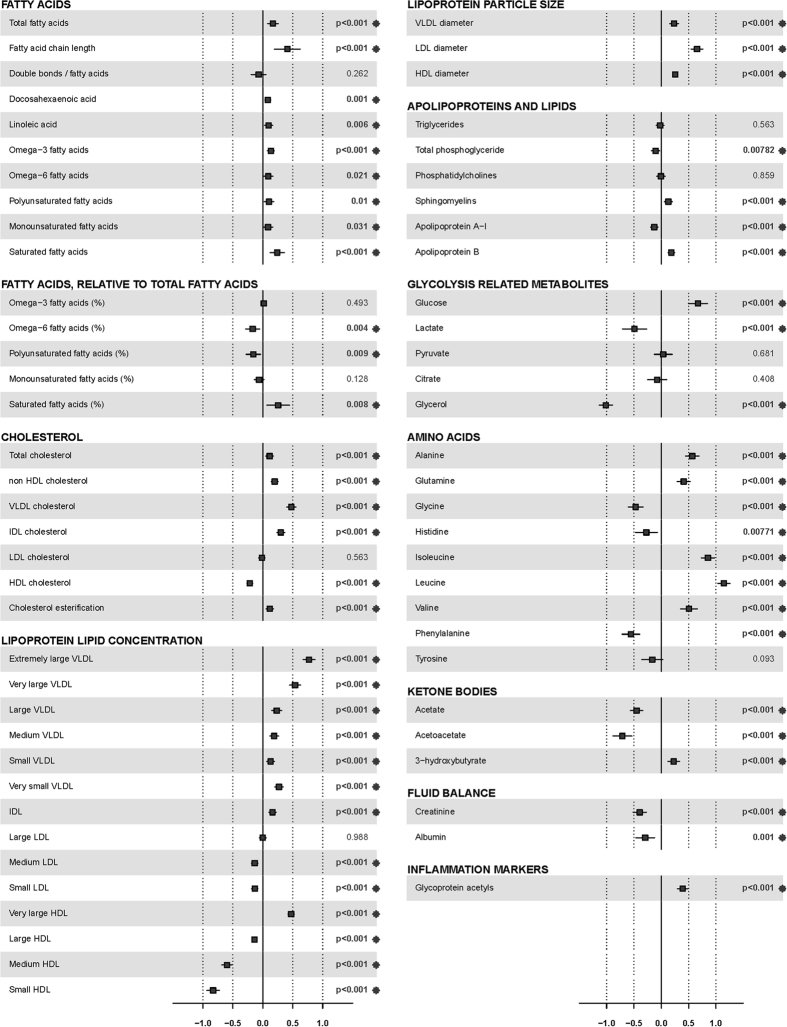
Intra-individual changes in metabolite levels after the standardized liquid meal. Effect sizes are indicated in units of 1-SD metabolite concentration and adjusted for age, sex, status, lipid-lowering medication and hypertension medication. Squares indicate β-regression coefficients and error bars denote 95% confidence intervals. Double bonds/fatty acids: the number of double bonds in fatty acids. VLDL, very low density lipoprotein; IDL, intermediate density lipoprotein; LDL, low density lipoprotein; HDL, high density lipoprotein. Asterisk behind the p-value indicates a significant association after FDR correction.

Thirty-five minutes after the SLM, almost all metabolites changed significantly compared to fasting levels (Fig. 1 and Supplementary Table S1). Especially LDL diameter, glucose, alanine and branched-chain amino acid levels increased, while glycerol, phenylalanine and acetoacetate levels decreased. Lipid concentration increased in the VLDL particles and decreased in most of the HDL particles. The levels of most fatty acids and lipids were minimally affected 33 minutes after the SLM. The magnitude of the effect of the SLM on metabolite levels ranged between a decrease of 1.0 SD (glycerol) to an increase of 1.1 SD (leucine). The effect of the SLM on the ^1^H-NMR metabolite levels was largely the same for all individuals.

### Association between BMI and metabolites is independent of fasting status

**Figure 2:**
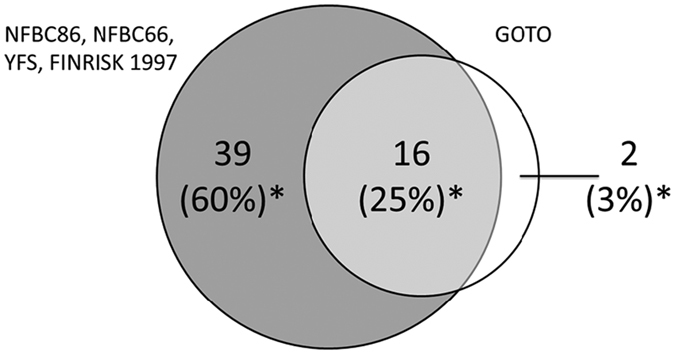
Number of metabolites associated with BMI in the studies in the article by Würtz *et al*.^6^ and the Growing Old Together (GOTO) study in fasting condition. Numbers indicate the number of metabolites significantly associated with BMI after correction for multiple testing. * % of the 65 metabolites measured in both studies. 8 metabolites (12%) were not associated with BMI in the GOTO study nor in the article by Würtz *et al*.^6^.

To investigate whether the association between metabolites and BMI was affected by fasting status, we performed regression analysis of metabolite levels with BMI in fasting status and after the SLM. In fasting status, 18 metabolite levels were significantly associated with BMI (Supplementary Table S2), of which 16 metabolites were also associated with BMI in the same direction as reported by Würtz *et al*.^6^ in a much larger study (N = 12,664) (Fig. 2, Supplementary Figure S1, Supplementary Table S3).

**Figure 3:**
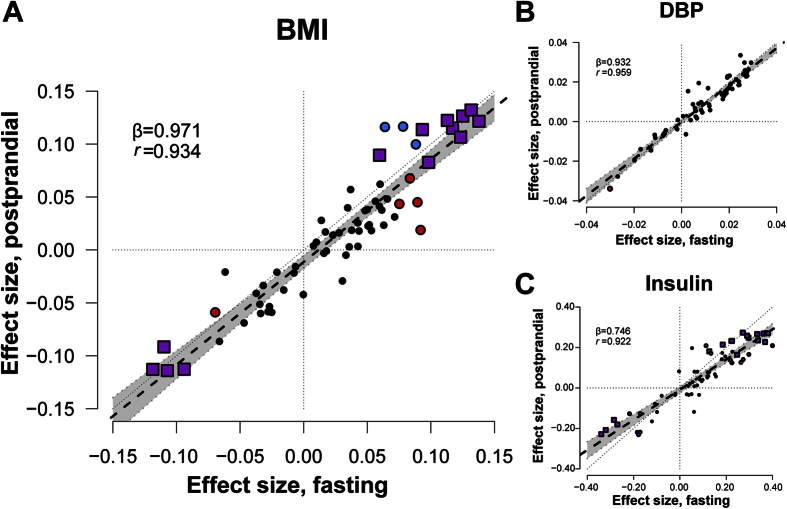
Effect size of the association between BMI, diastolic blood pressure (DBP) and insulin and metabolites before and after the standardized liquid meal. (**A**) BMI: Effect sizes indicate the association magnitudes in units of 1-SD metabolite concentration per 1-kg/m^2^ increment; (**B**) DBP: 1-SD metabolite concentration per 1-mm Hg increment; (**C**) Insulin: 1-SD metabolite concentration per 1-SD increment. Each dot represents a metabolite. Purple squares indicate the metabolite was significantly associated with BMI before and after the standardized liquid meal; Red circles: only significant in fasting samples; blue circles: only significant in postprandial samples; black circles: not significant in fasting and postprandial samples.

After the SLM, all metabolite levels showed highly similar effect size for their association with BMI as compared to the fasting state (Fig. 3A, Supplementary Figure S2, Supplementary Table S2), which is illustrated by the slope of the regression line of 0.971 (95% CI: 0.878–1.065) resembling the x = y diagonal. Despite the high similarity in effect sizes, we did observe some differences in the significances of the reported effects, for example extremely large VLDL and very large VLDL lipid concentration were significantly associated with BMI in the postprandial samples only. For any of the metabolites, however, we did not observe a significant association for the interaction between SLM and BMI (Supplementary Table S4).

### Association between other disease risk factors and metabolites is independent of fasting status

In order to generalize our conclusions to other disease risk factors, we performed the same analyses for diastolic blood pressure (DBP) and insulin levels. It should be noted that because there are no publications about the association between blood pressure and insulin levels with the Brainshake platform metabolites, we are not able to relate our finding to previous work. Nevertheless, we also observed for the associations of metabolite levels with DBP and insulin levels similar effect sizes in fasted status and after the SLM (DBP: *r* = 0.959; insulin: *r* = 0.922)(Fig. 3B and C; Supplementary Tables S5 and S6).

## Discussion

In this study we investigated how ^1^H-NMR metabolites in blood changed after a standardized liquid meal (SLM), which metabolites were associated with BMI and whether these associations were similar in fasting and postprandial status. Many metabolite levels changed within 35 minutes after the SLM. The strongest increases were observed for lipid concentration in large VLDL particles, LDL diameter, glucose and some amino acids, while the strongest decreases in metabolite levels were observed for glycerol and lipid concentration in small HDL particles. Levels of most fatty acids and lipids were minimally affected by SLM within 35 minutes. Despite the change in metabolite levels by the SLM, the association of metabolites with BMI remained similar. We concluded that SLM induces systematic changes in metabolite levels, but that the correlation structure between metabolite levels and BMI, as an example of a disease risk factor, is not affected by SLM.

The current study confirms the findings of a large association study between fasting metabolite levels and BMI^6^, and demonstrates that these associations are similar in fasting status and after a SLM. The strength of our study is that we compared the effect of fasting status on the association between metabolites and BMI in fasting and standardized non-fasting status in the same individuals.

Furthermore, we confirmed our findings with DBP and insulin levels in which we observed similar associations with metabolite levels in fasted status and after the SLM. Although the associations between metabolites and DBP were not significant, the effect sizes of the associations before and after the intervention were highly similar, indicated by the high correlation between the effect sizes (*r* = 0.959). Because insulin levels changed significantly after the SLM, we scaled insulin levels prior to the association analyses with metabolites. Without scaling (Supplementary Figure S3; Supplementary Table S6), effect sizes of the association between insulin and metabolite levels in fasted status were roughly 10 times larger as compared to the effect sizes in postprandial status. Interestingly, all changes in effect sizes were highly proportionate, as indicated by the near perfect correlation between effect size (*r* = 0.922 (Supplementary Figure S3)), indicating that the overall relation between insulin and metabolite levels is preserved. Thus, more research is required to generalize our findings, that the correlation structure between metabolite levels and BMI is not affected by SLM, for other health conditions or disease risk factors.

In spite of the relatively small study sample, the directions of the associations between metabolites and BMI were very similar to those observed by Würtz *et al*.^6^ (Supplementary Figure S1 and Supplementary Table S3). The observation that some metabolites showed significant association with BMI only in fasting or postprandial status may be due to limited power. Alternatively, for some metabolites postprandial status might inflate effect size of association. The strong decrease of glycerol and increase of VLDL cholesterol after SLM did, however, not affect the magnitude and direction of the association with BMI. Thus despite limited power, we are confident that our study is reliable, because the results of our study are in concordance with previous observations^6^.

That we tested the impact of a SLM after a standardized time may be considered as a limitation of our study. Usually, non-fasted study participants have had meals with variable content, portion size and variable times since the meal. The SLM mainly contains carbohydrates and therefore the association between metabolites and a disease risk factor might be different after a meal rich in fat or proteins for example. Therefore, the question remains how non-standardized meals and time after a meal affects associations of a disease risk factor with metabolite levels.

In conclusion, we showed that many ^1^H-NMR metabolites levels were influenced by a standardized liquid meal but that generally the associations between BMI and these metabolites remained largely unchanged in fasting status and after a SLM. Although our results on blood pressure and insulin levels support our findings, more research is required to investigate the generalizability of this observation to other disease risk factors and to uncontrolled non-fasting sampling. Good generalizability would then justify meta-analyses of cohorts irrespective of fasting status in meta-analyses associating serum metabolite levels and disease risk factors.

## Methods

### Study population

Inclusion and exclusion criteria for participants of this study have previously been described by van de Rest *et al*.^11^. In short, couples consisting of offspring from long-lived siblings and their current partners (controls) were included. In case one of the two was not eligible to participate, single offspring or controls were included to obtain the required sample size. In total we had 85 offspring of long-lived siblings and 68 controls, of which 56 couples. Participants were between 46 and 75 years, had a BMI between 23 and 35 kg/m^2^ and no diabetes (fasting glucose <7.0 mmol/L). The Medical Ethical Committee of the Leiden University Medical Center approved the study and all participants signed written informed consent. All experiments were performed in accordance with relevant and approved guidelines and regulations. This trial was registered at the Dutch Trial Register (http://www.trialregister.nl) as NTR3499.

### Study design

The measurements performed for this study were part of the Growing Old Together study^11^. This article only reports on the baseline measurements. The first blood collection was in fasting status and took place between 8 and 9 am in the hospital after at least 10 hours of fasting. The second blood collection was taken after on average 33 minutes after the standardized liquid meal (SLM) NutridrinkTM (range 20–42 minutes, SD 2 minutes) between 9 am and 12 am on the same day (Supplementary Figure S4). Nutridrink is a liquid oral nutritional supplement (Nutricia Advanced Medical Nutrition, Zoetermeer, The Netherlands; 1.5 kcal/mL, 35 En% fat, 49 En% carbohydrates, 16En% protein) (Supplementary Tables S7 and S8).

### Measurements

Height and weight were measured to the nearest 0.1 cm and 0.1 kg, respectively (Seca Clara 803, Seca Deutschland, Hamburg, Germany), with the person dressed in light clothing and without shoes. Insulin, measured before and after the SLM, was assessed in serum on the Immulite 2000 XPi (Siemens, Eschborn, Germany). Blood pressure measurements were calculated as the average of the four measurements and are described in detail by van de Rest *et al*. 11. Measurement of ^1^H-NMR metabolites is described in the article by Soininen *et al*.^12^. In the current study only metabolites analyzed by the study of Würtz *et al*.^6^ and van de Rest *et al*.^11^ were used for the analysis, resulting in 65 metabolites in total. All measurements were carried out in accordance with the relevant guidelines and regulations.

### Statistical analysis

For the following metabolites, serum levels were below the detection level for at least one measurement: lipid concentration in extremely large VLDL, very large VLDL, large VLDL, very large HDL and large HDL. To prevent these values to become missing after LN transformation, 1 was added to all values of these metabolites. All metabolite levels were LN transformed and standard normal-transformed. As insulin levels increased after the SLM, these levels were standard normal transformed.

The effect of the SLM on metabolite levels was determined using a linear mixed model with metabolites as outcome and adjusted for age, gender, status (longevity family member or control), lipid lowering medication, hypertension medication (fixed effects), household and individual (random effects). A random effect for household was included to account for the potentially increased similarity among household members (56 couples in our study), as they generally share diet and other lifestyle factors. A random effect for individual was included to pair the measurements before and after the SLM.

The association between the metabolites and BMI, insulin and DBP was determined using a linear regression model with metabolites as outcome and adjusted for age, gender, status (longevity family member or control), lipid lowering medication, hypertension medication (fixed effects) and household (random effects). To test whether the association between the metabolites and BMI was affected by the SLM, we added a variable defining the SLM and an interaction factor between SLM and BMI to this model. The Pearson correlation between the effect sizes in fasted status and after the SLM was calculated with a linear regression model.

All statistical analyses were performed with STATA ⁄SE 11.2 (StataCorp LP, College Station, TX, USA) and SPSS Statistics v23 (IBM Corp, Armonk, NY, USA). Associations significant after correction for false discovery rate (FDR) were considered significant.

## Additional Information

**How to cite this article**: Schutte, B. A. M. *et al*. The effect of standardized food intake on the association between BMI and ^1^H-NMR metabolites. *Sci. Rep.* **6**, 38980; doi: 10.1038/srep38980 (2016).

**Publisher's note:** Springer Nature remains neutral with regard to jurisdictional claims in published maps and institutional affiliations.

## Supplementary Material

Supplementary Information

## Figures and Tables

**Table 1:** Baseline characteristics of the study population.

Characteristic	N	Mean [range] (SD)
Mean age [range], years	153	62.9 [46.7–75.1]
Women, %	153	49.7
BMI, [range], kg/m^2^	153	26.9 [22.9–34.2]
Systolic BP, (SD), mm Hg	152	139.9 (17.3)
Diastolic BP, (SD), mm Hg	152	85.1 (8.0)
Total cholesterol, (SD), mmol/L	152	5.3 (1.0)
Fasting glucose, (SD), mmol/L	153	5.0 (0.6)
