# Supplementary material for: The effect of standardized food intake on the association between BMI and 1H-NMR metabolites
Source: Sci Rep. 2016 Dec 14;6:38980. doi: 10.1038/srep38980 (PMC5155225; doi:10.1038/srep38980)
Supplement: Supplementary Information [file srep38980-s1.pdf]

Supplementary Information for

The effect of standardized food intake on the association between BMI and ^{1}H-NMR metabolites

Bianca A.M. Schutte[1*], Erik B. van den Akker[1,2], Joris Deelen[1], Ondine van de Rest[3], Diana van Heemst[4], Edith J.M. Feskens[3] Marian Beekman[1#] and P. Eline Slagboom[1#*]

[1] *Department of Molecular Epidemiology, Leiden University Medical Center, 2300 RC Leiden, The Netherlands*

[2] *The Delft Bioinformatics Lab, Delft University of Technology, 2628 CD Delft, The Netherlands*

[3] *Division of Human Nutrition, Wageningen University, 6700 EV Wageningen, The Netherlands*

[4] *Department of Gerontology and Geriatrics, Leiden University Medical Center, 2300 RC Leiden, The Netherlands*

[#] *shared last author*

* *corresponding author*

Supplementary Figure S1│ Effect size of the association between BMI and metabolites in fasting status in this study and Würtz *et al.* (2014)[1]
Supplementary Figure S2│Effect size of the association between BMI and metabolites before and after the standardized liquid meal
Supplementary Figure S3│Effect size of the association between insulin and metabolites before and after the standardized liquid meal.
Supplementary Figure S4 │ Histogram of the time between consumption of the SLM and the collection of the non-fasted blood sample, in minutes.

Supplementary Table S1 │Changes in metabolite levels after the standardized liquid meal
Supplementary Table S2 │Effect size of the association between BMI and metabolites before and after the standardized liquid meal
Supplementary Table S3 │Effect size of the association between BMI and metabolites in this study and study done by Würtz *et al.* (2014)[1]
Supplementary Table S4 │Interaction between SLM and BMI
Supplementary Table S5 │Effect size of the association between diastolic blood pressure and metabolites before and after the standardized liquid meal
Supplementary Table S6 │Effect size of the association between insulin and metabolites before and after the standardized liquid meal
Supplementary Table S7│Nutritional content Nutridrink
Supplementary Table S8│Nutritional content amino acids Nutridrink

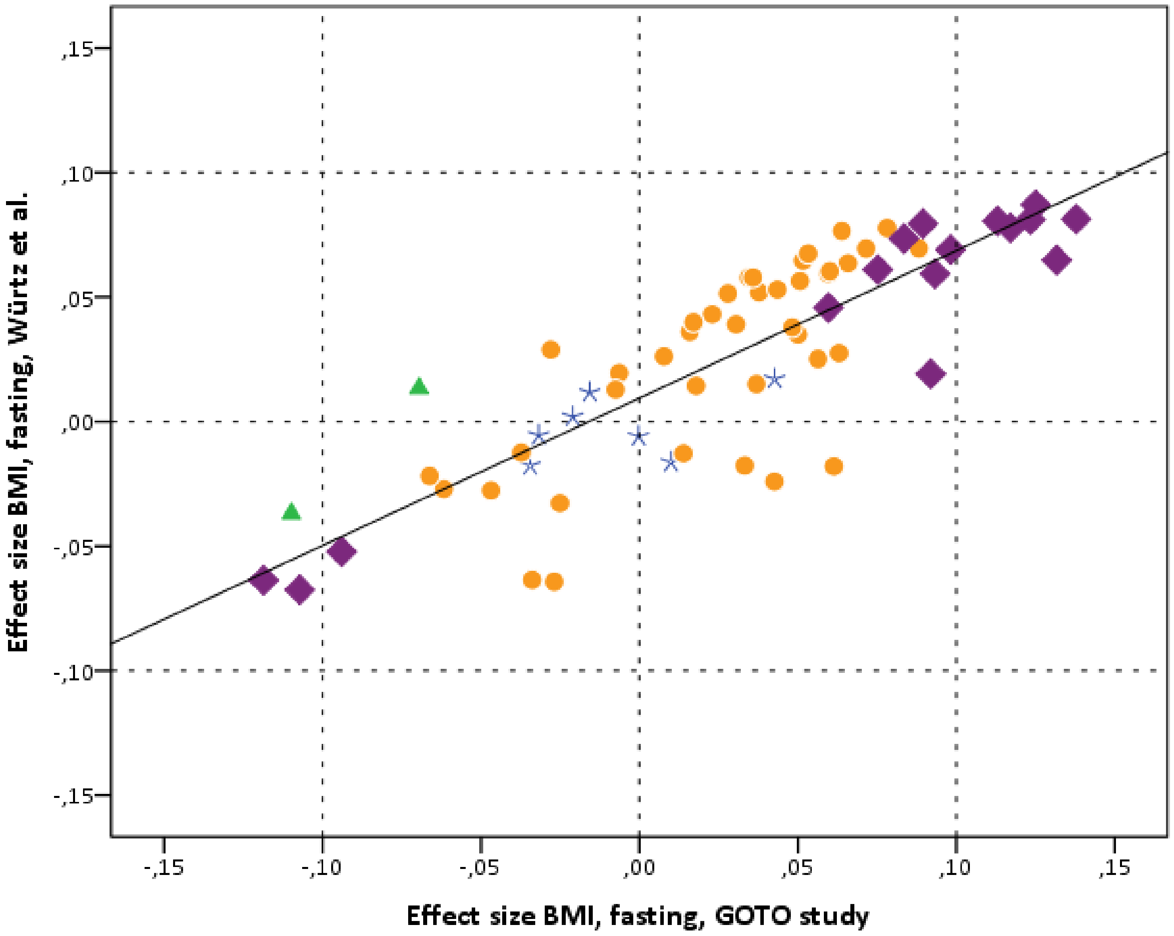

Supplementary Figure S1| Effect size of the association between BMI and metabolites in fasting status in this study and Würtz *et al.* (2014)[1]

Effect sizes indicate the association magnitudes in units of 1-SD metabolite concentration per 1-kg/m^2 increment in BMI. Each dot represents a metabolite. Purple diamonds indicate the metabolite was significantly associated with BMI in this study and Würtz *et al*.; green triangle: only significant in this study; orange circles: only significant in the study of Würtz *et al*; blue crosses: not significant in one of the studies

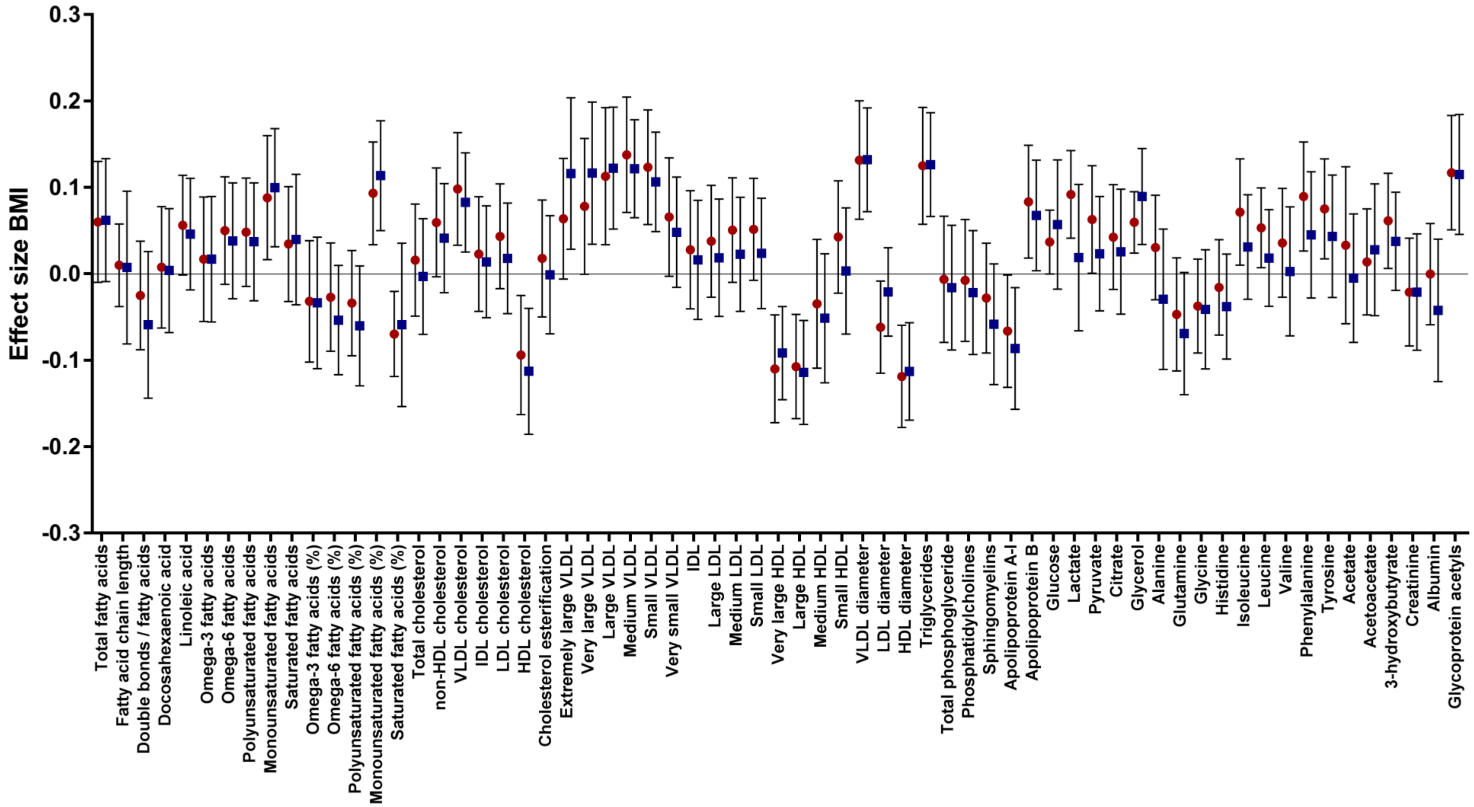

Supplementary Figure S2|Effect size of the association between BMI and metabolites before and after the standardized liquid meal

Effect sizes indicate the association magnitudes in units of 1-SD metabolite concentration per 1-kg/m^2 increment in BMI. Error bars indicate 95% confidence interval. Red circles indicate the effect size in fasting samples; blue squares indicate the effect size in postprandial samples. Double bonds/fatty acids: the number of double bonds in fatty acids. VLDL, very low density lipoprotein; IDL, intermediate density lipoprotein; LDL, low density lipoprotein; HDL, high density lipoprotein.

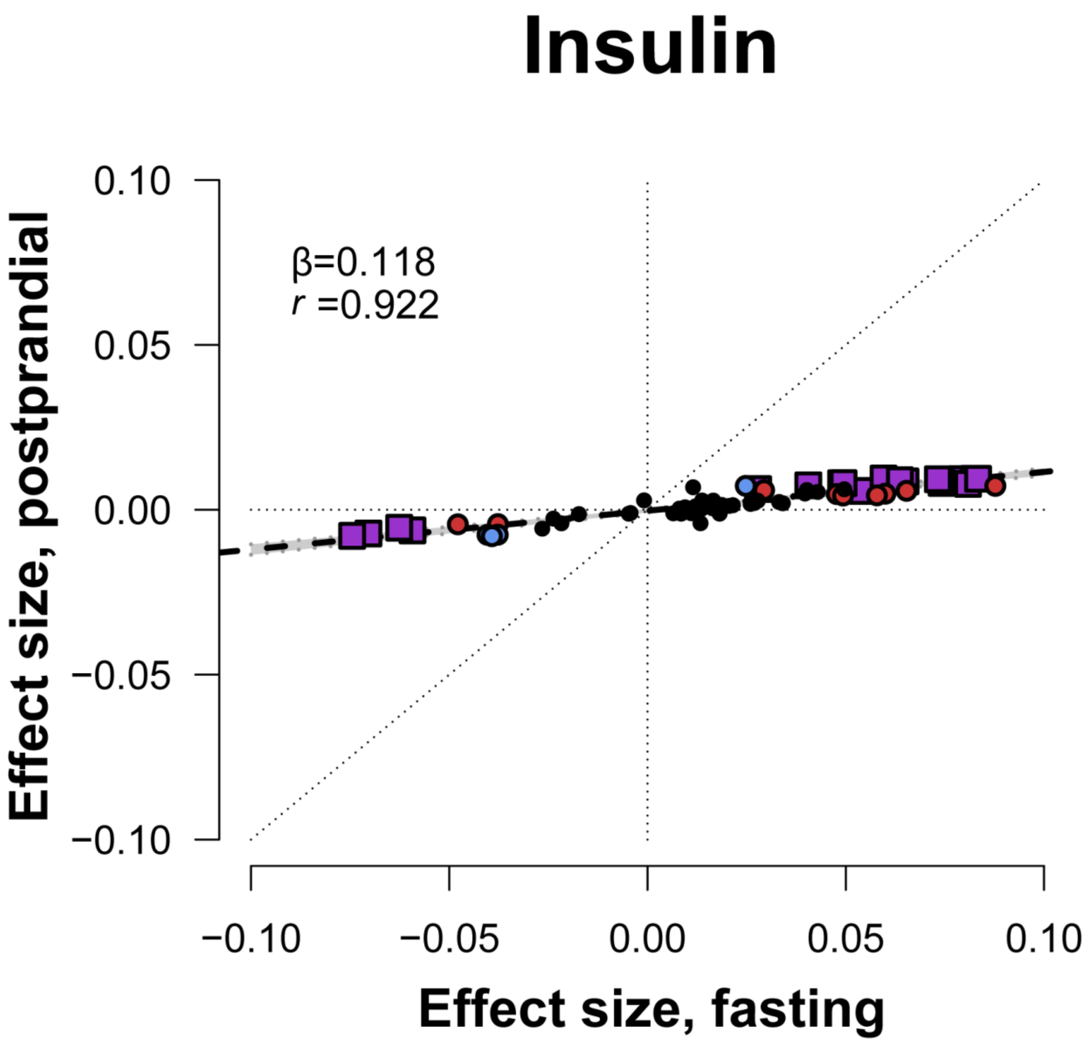

Supplementary Figure S3|Effect size of the association between insulin and metabolites before and after the standardized liquid meal.

Effect sizes indicate the association magnitudes in units of 1-SD metabolite concentration per 1-SD metabolite concentration per 1-MU/L increment in insulin. Each dot represents a metabolite. Purple squares indicate the metabolite was significantly associated with BMI before and after the standardized liquid meal; red circles: only significant in fasting samples; blue circles: only significant in postprandial samples; black circles: not significant in fasting and postprandial samples

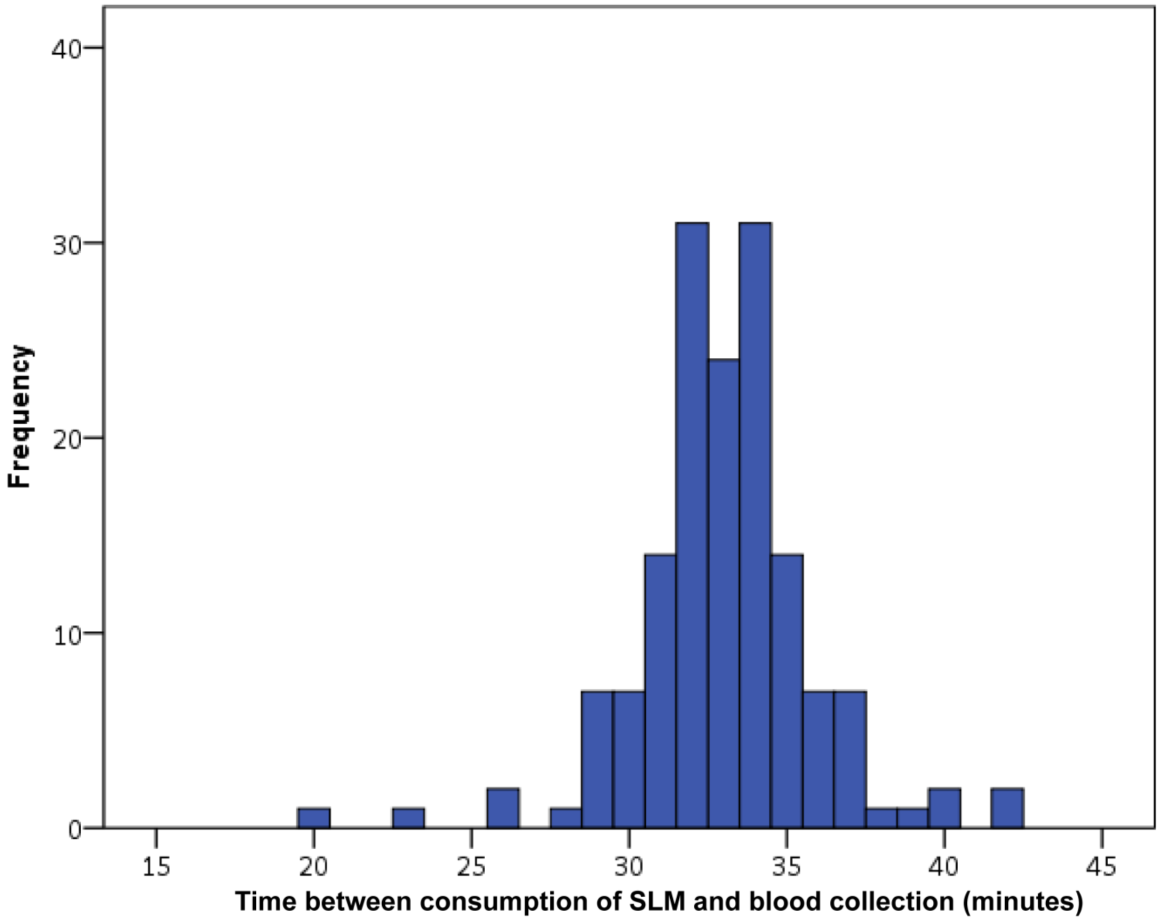

Supplementary Figure S4 | Histogram of the time between consumption of the SLM and the collection of the non-fasted blood sample, in minutes.

Supplementary Table S1 | Changes in metabolite levels after the standardized liquid meal

	N	Change	P-value*	95% CI	
**Fatty acids**					
Total fatty acids	146	0.169	**2.05E-04**	0.080	0.259
Fatty acid chain length	146	0.409	**1.80E-04**	0.195	0.624
Double bonds / fatty acids	146	-0.070	0.262	-0.192	0.052
Docosahexaenoic acid	146	0.083	**0.001**	0.034	0.132
Linoleic acid	146	0.095	**0.006**	0.027	0.163
Omega-3 fatty acids	146	0.135	**4.38E-06**	0.077	0.192
Omega-6 fatty acids	146	0.092	**0.021**	0.014	0.170
Polyunsaturated fatty acids	146	0.104	**0.010**	0.025	0.183
Monounsaturated fatty acids	146	0.087	**0.031**	0.008	0.166
Saturated fatty acids	146	0.241	**8.53E-05**	0.121	0.361
**Fatty acids, relative to total fatty acids**					
Omega-3 fatty acids (%)†	146	0.016	0.493	-0.030	0.063
Omega-6 fatty acids (%)†	146	-0.169	**0.004**	-0.284	-0.054
Polyunsaturated fatty acids (%)†	146	-0.159	**0.009**	-0.277	-0.040
Monounsaturated fatty acids (%)†	146	-0.063	0.128	-0.144	0.018
Saturated fatty acids (%)†	146	0.256	**0.008**	0.066	0.447
**Cholesterol**					
Total cholesterol	153	0.114	**4.22E-04**	0.051	0.177
Non-HDL cholesterol	153	0.198	**2.11E-11**	0.140	0.256
VLDL cholesterol	153	0.477	**6.08E-35**	0.401	0.552
IDL cholesterol	153	0.301	**6.32E-20**	0.237	0.366
LDL cholesterol	153	-0.016	0.563	-0.069	0.037
HDL cholesterol	153	-0.216	**7.77E-19**	-0.264	-0.169
Cholesterol esterification	146	0.116	**3.75E-04**	0.052	0.180
**Lipoprotein lipid concentration**					
Extremely large VLDL	149	0.772	**3.26E-52**	0.673	0.872
Very large VLDL	148	0.538	**1.55E-31**	0.448	0.629
Large VLDL	150	0.232	**4.09E-08**	0.149	0.315
Medium VLDL	153	0.190	**3.36E-07**	0.117	0.263
Small VLDL	153	0.131	**9.75E-05**	0.065	0.197
Very small VLDL	153	0.270	**9.75E-14**	0.199	0.341
IDL	153	0.162	**3.07E-07**	0.100	0.224
Large LDL	153	0.000	0.988	-0.056	0.056
Medium LDL	153	-0.135	**1.22E-06**	-0.190	-0.081
Small LDL	153	-0.134	**3.93E-07**	-0.185	-0.082
Very large HDL	153	0.473	**3.71E-76**	0.423	0.523
Large HDL	148	-0.137	**4.33E-12**	-0.176	-0.098
Medium HDL	153	-0.599	**2.58E-43**	-0.684	-0.514
Small HDL	153	-0.829	**4.35E-54**	-0.934	-0.724

Supplementary Table S1 (continued) |Changes in metabolite levels after the standardized liquid meal

	N	Change	P-value*	95% CI	
**Lipoprotein particle size**					
VLDL diameter	153	0.235	**1.43E-08**	0.154	0.316
LDL diameter	153	0.653	**3.47E-32**	0.545	0.762
HDL diameter	153	0.254	**1.99E-47**	0.219	0.288
**Apolipoproteins and lipids**					
Triglycerides	153	-0.021	0.563	-0.091	0.050
Total phosphoglyceride	146	-0.104	**7.82E-03**	-0.181	-0.027
Phosphatidylcholines	146	-0.007	0.859	-0.085	0.071
Sphingomyelins	146	0.129	**4.58E-04**	0.057	0.202
Apolipoprotein A-I	153	-0.128	**2.53E-04**	-0.196	-0.059
Apolipoprotein B	153	0.185	**2.24E-09**	0.125	0.246
**Glycolysis related metabolites**					
Glucose	153	0.672	**7.44E-14**	0.496	0.848
Lactate	153	-0.489	**1.52E-05**	-0.711	-0.268
Pyruvate	151	0.035	0.681	-0.131	0.201
Citrate	153	-0.074	0.408	-0.251	0.102
Glycerol	151	-1.014	**4.46E-60**	-1.135	-0.892
**Amino acids**					
Alanine	151	0.567	**1.94E-19**	0.444	0.690
Glutamine	151	0.410	**2.51E-11**	0.289	0.530
Glycine	135	-0.468	**1.79E-11**	-0.604	-0.331
Histidine	153	-0.273	**0.00771**	-0.473	-0.072
Isoleucine	150	0.853	**1.9E-42**	0.731	0.976
Leucine	152	1.146	**3.15E-89**	1.034	1.258
Valine	151	0.506	**7.93E-11**	0.354	0.659
Phenylalanine	153	-0.554	**1.28E-11**	-0.714	-0.394
Tyrosine	153	-0.165	0.093	-0.358	0.027
**Ketone bodies**					
Acetate	153	-0.452	**1.62E-14**	-0.567	-0.336
Acetoacetate	153	-0.711	**6.66E-16**	-0.884	-0.538
3-hydroxybutyrate	151	0.227	**3.28E-05**	0.120	0.334
**Fluid balance**					
Creatinine	152	-0.394	**1.55E-10**	-0.515	-0.273
Albumin	153	-0.294	**0.001**	-0.466	-0.122
**Inflammation markers**					
Glycoprotein acetyls	153	0.389	**2.22E-16**	0.296	0.483

Changes are in units of 1-SD metabolite concentration

* Bold p-values indicate significant associations after FDR correction

† % of total fatty acids

Double bonds/fatty acids: the number of double bonds in fatty acids. VLDL, very low density lipoprotein; IDL, intermediate density lipoprotein; LDL, low density lipoprotein; HDL, high density lipoprotein.

Supplementary Table S2 |Effect size of the association between BMI and metabolites before and after the standardized liquid meal

		fasting				postprandial			
	N	B	p-value*	95% CI		B	p-value*	95% CI	
**Fatty acids**									
Total fatty acids	146	0.060	0.091	-0.010	0.130	0.062	0.086	-0.009	0.133
Fatty acid chain length	146	0.010	0.679	-0.038	0.058	0.007	0.870	-0.081	0.096
Double bonds / fatty acids	146	-0.025	0.430	-0.088	0.038	-0.059	0.171	-0.144	0.026
Docosahexaenoic acid	146	0.008	0.828	-0.063	0.078	0.004	0.915	-0.068	0.076
Linoleic acid	146	0.056	0.055	-0.001	0.114	0.046	0.161	-0.019	0.111
Omega-3 fatty acids	146	0.017	0.640	-0.055	0.089	0.017	0.643	-0.056	0.090
Omega-6 fatty acids	146	0.050	0.114	-0.012	0.112	0.038	0.261	-0.029	0.105
Polyunsaturated fatty acids	146	0.048	0.130	-0.014	0.111	0.037	0.282	-0.031	0.105
Monounsaturated fatty acids	146	0.088	0.016	0.017	0.160	0.100	**0.005**	0.031	0.168
Saturated fatty acids	146	0.035	0.304	-0.032	0.101	0.040	0.298	-0.036	0.115
**Fatty acids, relative to total fatty acids**									
Omega-3 fatty acids (%)[†]	146	-0.032	0.371	-0.102	0.038	-0.034	0.384	-0.110	0.043
Omega-6 fatty acids (%)[†]	146	-0.027	0.396	-0.089	0.036	-0.054	0.096	-0.117	0.010
Polyunsaturated fatty acids (%)[†]	146	-0.034	0.273	-0.095	0.027	-0.060	0.089	-0.129	0.009
Monounsaturated fatty acids (%)[†]	146	0.093	**0.002**	0.034	0.153	0.114	**0.001**	0.050	0.177
Saturated fatty acids (%)[†]	146	-0.070	**0.006**	-0.119	-0.020	-0.059	0.218	-0.153	0.035
**Cholesterol**									
Total cholesterol	153	0.016	0.628	-0.049	0.081	-0.003	0.926	-0.070	0.064
Non-HDL cholesterol	153	0.060	0.064	-0.003	0.123	0.041	0.197	-0.022	0.104
VLDL cholesterol	153	0.098	**0.004**	0.033	0.163	0.083	**0.005**	0.025	0.140
IDL cholesterol	153	0.023	0.493	-0.043	0.089	0.014	0.670	-0.051	0.079
LDL cholesterol	153	0.044	0.158	-0.017	0.104	0.018	0.580	-0.046	0.082
HDL cholesterol	153	-0.094	**0.008**	-0.163	-0.025	-0.113	**0.003**	-0.186	-0.040
Cholesterol esterification	146	0.018	0.600	-0.050	0.086	-0.001	0.976	-0.069	0.067

Supplementary Table S2 (continued)|Effect size of the association between BMI and metabolites before and after the standardized liquid meal

		fasting				postprandial			
	N	B	p-value*	95% CI		B	p-value*	95% CI	
**Lipoprotein lipid concentration**									
Extremely large VLDL	149	0.064	0.073	-0.006	0.134	0.116	**0.010**	0.028	0.204
Very large VLDL	148	0.078	0.051	0.000	0.157	0.117	**0.006**	0.034	0.199
Large VLDL	150	0.113	**0.006**	0.034	0.192	0.122	**0.001**	0.052	0.193
Medium VLDL	153	0.138	**8.81E-05**	0.071	0.205	0.122	**4.73E-05**	0.065	0.178
Small VLDL	153	0.123	**3.59E-04**	0.057	0.190	0.107	**3.90E-04**	0.049	0.164
Very small VLDL	153	0.066	0.059	-0.003	0.134	0.048	0.138	-0.016	0.112
IDL	153	0.028	0.420	-0.040	0.096	0.016	0.643	-0.053	0.085
Large LDL	153	0.038	0.249	-0.027	0.102	0.019	0.588	-0.049	0.087
Medium LDL	153	0.051	0.099	-0.010	0.111	0.023	0.499	-0.043	0.089
Small LDL	153	0.052	0.086	-0.007	0.111	0.024	0.463	-0.040	0.088
Very large HDL	153	-0.110	**0.001**	-0.172	-0.047	-0.092	**0.001**	-0.146	-0.038
Large HDL	148	-0.107	**0.001**	-0.168	-0.047	-0.114	**2.95E-04**	-0.174	-0.054
Medium HDL	153	-0.034	0.361	-0.109	0.040	-0.051	0.176	-0.126	0.023
Small HDL	153	0.043	0.196	-0.022	0.108	0.003	0.928	-0.070	0.076
**Lipoprotein particle size**									
VLDL diameter	153	0.132	**2.43E-04**	0.063	0.200	0.132	**3.16E-05**	0.072	0.192
LDL diameter	153	-0.062	0.024	-0.115	-0.008	-0.021	0.419	-0.072	0.030
HDL diameter	153	-0.119	**1.35E-04**	-0.178	-0.059	-0.113	**1.40E-04**	-0.169	-0.056
**Apolipoproteins and lipids**									
Triglycerides	153	0.125	**3.89E-04**	0.058	0.193	0.126	**6.58E-05**	0.066	0.187
Total phosphoglyceride	146	-0.006	0.862	-0.079	0.067	-0.016	0.663	-0.088	0.056
Phosphatidylcholines	146	-0.008	0.833	-0.078	0.063	-0.022	0.549	-0.093	0.050
Sphingomyelins	146	-0.028	0.383	-0.092	0.035	-0.058	0.100	-0.128	0.011
Apolipoprotein A-I	153	-0.066	0.046	-0.131	-0.001	-0.086	0.017	-0.157	-0.016
Apolipoprotein B	153	0.083	**0.013**	0.018	0.149	0.068	0.038	0.004	0.132

Supplementary Table S2 (continued)|Effect size of the association between BMI and metabolites before and after the standardized liquid meal

		fasting				postprandial			
	N	B	p-value*	95% CI		B	p-value*	95% CI	
**Glycolysis related metabolites**									
Glucose	153	0.037	0.050	0.000	0.074	0.057	0.133	-0.018	0.132
Lactate	153	0.092	**0.001**	0.041	0.143	0.019	0.659	-0.066	0.103
Pyruvate	151	0.063	0.047	0.001	0.125	0.023	0.485	-0.043	0.090
Citrate	153	0.043	0.167	-0.018	0.103	0.026	0.484	-0.047	0.098
Glycerol	151	0.060	**0.001**	0.024	0.095	0.089	**0.002**	0.034	0.145
**Amino acids**									
Alanine	151	0.030	0.319	-0.030	0.091	-0.029	0.475	-0.110	0.052
Glutamine	151	-0.047	0.159	-0.112	0.019	-0.069	0.056	-0.140	0.002
Glycine	135	-0.037	0.177	-0.092	0.017	-0.041	0.240	-0.110	0.028
Histidine	153	-0.016	0.575	-0.071	0.040	-0.038	0.219	-0.099	0.023
Isoleucine	150	0.072	0.023	0.010	0.133	0.031	0.308	-0.029	0.092
Leucine	152	0.053	0.024	0.007	0.099	0.018	0.518	-0.038	0.074
Valine	151	0.036	0.262	-0.027	0.099	0.003	0.939	-0.072	0.078
Phenylalanine	153	0.089	**0.006**	0.026	0.153	0.045	0.224	-0.028	0.118
Tyrosine	153	0.075	**0.011**	0.017	0.133	0.044	0.226	-0.027	0.114
**Ketone bodies**									
Acetate	153	0.033	0.470	-0.058	0.124	-0.005	0.896	-0.079	0.069
Acetoacetate	153	0.014	0.654	-0.047	0.075	0.028	0.469	-0.048	0.104
3-hydroxybutyrate	151	0.061	0.029	0.006	0.116	0.038	0.191	-0.019	0.094
**Fluid balance**									
Creatinine	152	-0.021	0.504	-0.083	0.041	-0.021	0.536	-0.088	0.046
Albumin	153	0.000	0.993	-0.059	0.058	-0.042	0.312	-0.125	0.040
**Inflammation markers**									
Glycoprotein acetyls	153	0.117	**0.001**	0.051	0.183	0.115	**0.001**	0.046	0.184

Effect sizes indicate the association magnitudes in units of 1-SD metabolite concentration per 1-kg/m^2 increment in BMI
* Bold p-values indicate significant associations after FDR correction. † % of total fatty acids
Double bonds/fatty acids: the number of double bonds in fatty acids. VLDL, very low density lipoprotein; IDL, intermediate density lipoprotein; LDL, low density lipoprotein; HDL, high density lipoprotein.

Supplementary Table S3 | Effect size of the association between BMI and metabolites in this study and study done by Würtz *et al.* (2014)[1]

	GOTO			Würtz		
	B	SE	p-value	B	SE	p-value
**Fatty acids**						
Total fatty acids	0.060	0.035	0.091	0.060	0.003	**1.74E-71**
Fatty acid chain length	0.010	0.024	0.679	-0.016	0.006	6.70E-03
Double bonds / fatty acids	-0.025	0.032	0.430	-0.033	0.004	**8.51E-17**
Docosahexaenoic acid	0.008	0.035	0.828	0.026	0.005	**3.90E-07**
Linoleic acid	0.056	0.029	0.055	0.025	0.004	**3.24E-10**
Omega-3 fatty acids	0.017	0.036	0.640	0.040	0.006	**9.50E-13**
Omega-6 fatty acids	0.050	0.031	0.114	0.035	0.005	**5.36E-14**
Polyunsaturated fatty acids	0.048	0.032	0.130	0.038	0.005	**5.83E-15**
Monounsaturated fatty acids	0.088	0.036	0.016	0.070	0.003	**2.37E-145**
Saturated fatty acids	0.035	0.033	0.304	0.058	0.003	**2.52E-102**
**Fatty acids, relative to total fatty acids**						
Omega-3 fatty acids (%)†	-0.032	0.035	0.371	-0.005	0.004	0.135
Omega-6 fatty acids (%)†	-0.027	0.032	0.396	-0.064	0.003	**1.03E-117**
Polyunsaturated fatty acids (%)†	-0.034	0.031	0.273	-0.064	0.004	**1.13E-71**
Monounsaturated fatty acids (%)†	0.093	0.030	**0.002**	0.059	0.002	**1.88E-159**
Saturated fatty acids (%)†	-0.070	0.025	**0.006**	0.013	0.004	8.95E-04
**Cholesterol**						
Total cholesterol	0.016	0.033	0.628	0.036	0.003	**2.58E-30**
non HDL cholesterol	0.060	0.032	0.064	0.060	0.003	**3.07E-107**
VLDL cholesterol	0.098	0.033	**0.004**	0.069	0.003	**8.41E-110**
IDL cholesterol	0.023	0.033	0.493	0.043	0.003	**7.44E-49**
LDL cholesterol	0.044	0.031	0.158	0.053	0.002	**1.47E-116**
HDL cholesterol	-0.094	0.035	**0.008**	-0.052	0.002	**1.72E-133**
Cholesterol esterification	0.018	0.034	0.600	0.014	0.003	**2.17E-05**
**Lipoprotein lipid concentration**						
Extremely large VLDL	0.064	0.035	0.073	0.077	0.002	**8.74E-287**
Very large VLDL	0.078	0.040	0.051	0.078	0.002	**2.43E-231**
Large VLDL	0.113	0.040	**0.006**	0.081	0.002	**3.66E-294**
Medium VLDL	0.138	0.034	**8.81E-05**	0.081	0.003	**4.68E-163**
Small VLDL	0.123	0.033	**3.59E-04**	0.081	0.004	**2.95E-114**
Very small VLDL	0.066	0.034	0.059	0.064	0.002	**1.15E-188**
IDL	0.028	0.034	0.420	0.051	0.003	**1.24E-90**
Large LDL	0.038	0.033	0.249	0.052	0.002	**6.12E-120**
Medium LDL	0.051	0.030	0.099	0.056	0.002	**4.25E-149**
Small LDL	0.052	0.030	0.086	0.065	0.002	**2.20E-202**
Very large HDL	-0.110	0.031	**0.001**	-0.037	0.013	5.49E-03
Large HDL	-0.107	0.030	**0.001**	-0.068	0.002	**3.93E-185**
Medium HDL	-0.034	0.038	0.361	-0.018	0.010	0.079
Small HDL	0.043	0.033	0.196	0.017	0.012	0.151

Supplementary Table S3 (continued)|Effect size of the association between BMI and metabolites in this study and study done by Würtz *et al.* (2014)[1]

	GOTO			Würtz		
	B	SE	p-value	B	SE	p-value
**Lipoprotein particle size**						
VLDL diameter	0.132	0.035	**2.43E-04**	0.065	0.002	**3.74E-206**
LDL diameter	-0.062	0.027	0.024	-0.027	0.003	**2.38E-21**
HDL diameter	-0.119	0.030	**1.35E-04**	-0.064	0.009	**1.33E-11**
**Apolipoproteins and lipids**						
Triglycerides	0.125	0.034	**3.89E-04**	0.087	0.002	**0**
Total phosphoglyceride	-0.006	0.037	0.862	0.020	0.003	**5.14E-12**
Phosphatidylcholines	-0.008	0.036	0.833	0.013	0.003	**2.54E-06**
Sphingomyelins	-0.028	0.032	0.383	0.029	0.003	**3.03E-30**
Apolipoprotein A-I	-0.066	0.033	0.046	-0.022	0.003	**2.27E-14**
Apolipoprotein B	0.083	0.033	**0.013**	0.073	0.003	**3.68E-100**
**Glycolysis related metabolites**						
Glucose	0.037	0.019	0.050	0.015	0.004	**1.97E-05**
Lactate	0.092	0.026	**0.001**	0.019	0.003	**2.59E-10**
Pyruvate	0.063	0.031	0.047	0.028	0.005	**2.17E-08**
Citrate	0.043	0.031	0.167	-0.024	0.006	**2.32E-05**
Glycerol	0.060	0.018	**0.001**	0.046	0.007	**5.88E-11**
**Amino acids**						
Alanine	0.030	0.030	0.319	0.039	0.004	**1.06E-24**
Glutamine	-0.047	0.033	0.159	-0.028	0.005	**1.08E-07**
Glycine	-0.037	0.027	0.177	-0.012	0.004	**5.38E-04**
Histidine	-0.016	0.028	0.575	0.012	0.007	0.100
Isoleucine	0.072	0.031	0.023	0.070	0.003	**1.92E-105**
Leucine	0.053	0.023	0.024	0.067	0.002	**7.12E-249**
Valine	0.036	0.032	0.262	0.058	0.002	**3.70E-164**
Phenylalanine	0.089	0.032	**0.006**	0.079	0.002	**2.00E-292**
Tyrosine	0.075	0.029	**0.011**	0.061	0.004	**1.12E-60**
**Ketone bodies**						
Acetate	0.033	0.046	0.470	-0.018	0.002	**1.92E-14**
Acetoacetate	0.014	0.031	0.654	-0.013	0.002	**3.02E-08**
3-hydroxybutyrate	0.061	0.028	0.029	-0.018	0.002	**5.20E-15**
**Fluid balance**						
Creatinine	-0.021	0.031	0.504	0.002	0.007	0.776
Albumin	0.000	0.030	0.993	-0.006	0.005	0.196
**Inflammation markers**						
Glycoprotein acetyls	0.117	0.033	**0.001**	0.078	0.005	**4.67E-53**

Effect sizes indicate the association magnitudes in units of 1-SD metabolite concentration per 1-kg/m^2 increment in BMI

* Bold p-values indicate significant associations after FDR correction. # Bold p-values indicate significant associations after Bonferroni correction. † % of total fatty acids

Double bonds/fatty acids: the number of double bonds in fatty acids. VLDL. very low density lipoprotein; IDL. intermediate density lipoprotein; LDL. low density lipoprotein; HDL. high density lipoprotein.

Supplementary Table S4 |Interaction between SLM and BMI

		BMI				SLM x BMI				SLM			
	N	B	p-value*	95% CI		B	p-value*	95% CI		B	p-value*	95% CI	
**Fatty acids**													
Total fatty acids	146	0.060	0.089	-0.009	0.130	0.002	0.919	-0.034	0.037	0.121	0.808	-0.861	1.103
Fatty acid chain length	146	0.021	0.385	-0.027	0.070	-0.026	0.631	-0.131	0.080	1.099	0.453	-1.794	3.992
Double bonds / fatty acids	146	-0.030	0.343	-0.092	0.032	-0.024	0.454	-0.088	0.040	0.581	0.513	-1.178	2.340
Docosahexaenoic acid	146	0.008	0.828	-0.062	0.078	-0.004	0.716	-0.024	0.017	0.184	0.519	-0.381	0.749
Linoleic acid	146	0.055	0.060	-0.002	0.113	-0.008	0.591	-0.036	0.021	0.305	0.442	-0.479	1.089
Omega-3 fatty acids	146	0.015	0.671	-0.056	0.087	0.003	0.802	-0.022	0.029	0.048	0.891	-0.645	0.740
Omega-6 fatty acids	146	0.047	0.135	-0.015	0.109	-0.006	0.708	-0.039	0.026	0.258	0.566	-0.630	1.145
Polyunsaturated fatty acids	146	0.045	0.154	-0.017	0.108	-0.005	0.748	-0.038	0.028	0.248	0.585	-0.650	1.146
Monounsaturated fatty acids	146	0.086	0.018	0.015	0.157	0.015	0.312	-0.015	0.046	-0.329	0.432	-1.159	0.500
Saturated fatty acids	146	0.039	0.249	-0.027	0.105	-0.003	0.920	-0.056	0.050	0.314	0.674	-1.161	1.788
**Fatty acids, relative to total fatty acids**													
Omega-3 fatty acids (%)[†]	146	-0.034	0.339	-0.104	0.036	0.002	0.853	-0.023	0.028	-0.049	0.891	-0.754	0.656
Omega-6 fatty acids (%)[†]	146	-0.033	0.301	-0.095	0.030	-0.015	0.615	-0.075	0.045	0.240	0.773	-1.410	1.890
Polyunsaturated fatty acids (%)[†]	146	-0.040	0.195	-0.100	0.021	-0.014	0.647	-0.077	0.048	0.229	0.792	-1.490	1.947
Monounsaturated fatty acids (%)[†]	146	0.089	**0.004**	0.030	0.148	0.029	0.058	-0.001	0.058	-0.834	0.044	-1.647	-0.022
Saturated fatty acids (%)[†]	146	-0.057	0.024	-0.107	-0.008	-0.014	0.770	-0.106	0.078	0.622	0.628	-1.917	3.161
**Cholesterol**													
Total cholesterol	153	0.015	0.647	-0.050	0.080	-0.017	0.194	-0.044	0.009	0.579	0.107	-0.128	1.286
non-HDL cholesterol	153	0.058	0.070	-0.005	0.121	-0.015	0.211	-0.039	0.009	0.600	0.064	-0.037	1.237
VLDL cholesterol	153	0.095	**0.004**	0.030	0.159	-0.009	0.580	-0.040	0.022	0.711	0.105	-0.152	1.575
IDL cholesterol	153	0.022	0.505	-0.044	0.089	-0.008	0.569	-0.036	0.020	0.516	0.173	-0.231	1.262
LDL cholesterol	153	0.043	0.163	-0.018	0.103	-0.024	0.032	-0.045	-0.002	0.621	0.035	0.044	1.199
HDL cholesterol	153	-0.093	**0.009**	-0.162	-0.024	-0.021	0.021	-0.039	-0.003	0.345	0.163	-0.142	0.833
Cholesterol esterification	146	0.017	0.627	-0.051	0.084	-0.016	0.226	-0.043	0.010	0.556	0.131	-0.168	1.280

Supplementary Table S4 (continued)|Interaction between SLM and BMI

	BMI					BMI x SLM				SLM			
	N	B	p-value*	95% CI		B	p-value*	95% CI		B	p-value*	95% CI	
**Lipoprotein lipid concentration**													
Extremely large VLDL	149	0.062	0.080	-0.008	0.132	0.056	0.006	0.016	0.096	-0.741	0.176	-1.820	0.338
Very large VLDL	148	0.076	0.054	-0.001	0.153	0.043	0.010	0.010	0.075	-0.613	0.174	-1.501	0.275
Large VLDL	150	0.110	**0.006**	0.032	0.188	0.015	0.290	-0.013	0.044	-0.184	0.649	-0.982	0.615
Medium VLDL	153	0.134	**1.21E-04**	0.067	0.200	-0.008	0.579	-0.035	0.020	0.396	0.297	-0.355	1.148
Small VLDL	153	0.119	**4.96E-04**	0.054	0.185	-0.008	0.507	-0.033	0.016	0.354	0.300	-0.321	1.029
Very small VLDL	153	0.063	0.069	-0.005	0.132	-0.013	0.402	-0.043	0.017	0.613	0.141	-0.208	1.434
IDL	153	0.027	0.439	-0.042	0.095	-0.009	0.477	-0.036	0.017	0.417	0.245	-0.291	1.124
Large LDL	153	0.036	0.263	-0.028	0.101	-0.017	0.155	-0.039	0.006	0.444	0.154	-0.170	1.058
Medium LDL	153	0.049	0.103	-0.010	0.109	-0.026	0.030	-0.049	-0.002	0.554	0.077	-0.062	1.171
Small LDL	153	0.051	0.088	-0.008	0.109	-0.026	0.020	-0.048	-0.004	0.563	0.055	-0.012	1.138
Very large HDL	153	-0.109	**0.001**	-0.171	-0.047	0.016	0.184	-0.008	0.039	0.047	0.883	-0.591	0.686
Large HDL	148	-0.107	**0.001**	-0.168	-0.046	-0.007	0.344	-0.022	0.008	0.058	0.782	-0.358	0.474
Medium HDL	153	-0.035	0.362	-0.109	0.040	-0.017	0.326	-0.050	0.017	-0.151	0.745	-1.072	0.770
Small HDL	153	0.045	0.168	-0.019	0.110	-0.045	0.028	-0.084	-0.005	0.369	0.504	-0.724	1.463
**Lipoprotein particle size**													
VLDL diameter	153	0.128	**2.83E-04**	0.061	0.196	0.007	0.640	-0.023	0.038	0.039	0.926	-0.803	0.882
LDL diameter	153	-0.062	0.024	-0.116	-0.008	0.042	0.116	-0.010	0.094	-0.471	0.505	-1.868	0.927
HDL diameter	153	-0.120	**1.12E-04**	-0.179	-0.061	0.008	0.291	-0.007	0.023	0.038	0.855	-0.372	0.448
**Apolipoproteins and lipids**													
Triglycerides	153	0.121	**0.001**	0.054	0.187	0.010	0.455	-0.016	0.036	-0.288	0.426	-1.003	0.427
Total phosphoglyceride	146	-0.008	0.826	-0.081	0.065	-0.006	0.713	-0.039	0.027	0.059	0.897	-0.839	0.956
Phosphatidylcholines	146	-0.009	0.800	-0.080	0.062	-0.011	0.496	-0.044	0.021	0.293	0.514	-0.596	1.183
Sphingomyelins	146	-0.030	0.353	-0.094	0.034	-0.026	0.115	-0.059	0.007	0.840	0.066	-0.057	1.736
Apolipoprotein A-I	153	-0.065	0.049	-0.131	0.000	-0.022	0.124	-0.050	0.006	0.462	0.238	-0.311	1.236
Apolipoprotein B	153	0.081	0.015	0.016	0.145	-0.010	0.384	-0.034	0.013	0.468	0.154	-0.179	1.114

Supplementary Table S4 (continued) | Interaction between SLM and BMI

	BMI					SLM x BMI				SLM			
	N	B	p-value*	95% CI		B	p-value*	95% CI		B	p-value*	95% CI	
**Glycolysis related metabolites**													
Glucose	153	0.031	0.146	-0.011	0.074	0.031	0.339	-0.033	0.096	-0.174	0.849	-1.983	1.634
Lactate	153	0.085	**0.001**	0.034	0.135	-0.058	0.179	-0.144	0.027	1.081	0.359	-1.248	3.410
Pyruvate	151	0.057	0.082	-0.007	0.121	-0.027	0.425	-0.094	0.040	0.763	0.398	-1.022	2.547
Citrate	153	0.045	0.141	-0.015	0.104	-0.021	0.573	-0.095	0.053	0.492	0.630	-1.533	2.517
Glycerol	151	0.065	**4.77E-04**	0.029	0.100	0.020	0.453	-0.033	0.073	-1.551	0.038	-3.013	-0.089
**Amino acids**													
Alanine	151	0.027	0.383	-0.034	0.088	-0.053	0.056	-0.107	0.001	1.992	0.007	0.557	3.428
Glutamine	151	-0.044	0.176	-0.108	0.020	-0.028	0.362	-0.089	0.033	1.164	0.161	-0.472	2.800
Glycine	135	-0.036	0.224	-0.094	0.022	-0.007	0.812	-0.064	0.050	-0.285	0.719	-1.849	1.280
Histidine	153	-0.011	0.680	-0.065	0.043	-0.031	0.441	-0.110	0.048	0.561	0.613	-1.631	2.753
Isoleucine	150	0.064	0.040	0.003	0.126	-0.026	0.319	-0.077	0.025	1.549	0.030	0.151	2.946
Leucine	152	0.047	0.050	0.000	0.095	-0.023	0.356	-0.073	0.026	1.769	0.011	0.423	3.116
Valine	151	0.030	0.349	-0.034	0.094	-0.022	0.449	-0.079	0.035	1.097	0.168	-0.469	2.664
Phenylalanine	153	0.081	0.013	0.018	0.145	-0.028	0.424	-0.099	0.042	0.212	0.826	-1.698	2.122
Tyrosine	153	0.071	0.021	0.011	0.131	-0.023	0.548	-0.097	0.052	0.445	0.674	-1.648	2.538
**Ketone bodies**													
Acetate	153	0.027	0.561	-0.064	0.118	-0.025	0.305	-0.074	0.023	0.226	0.736	-1.099	1.550
Acetoacetate	153	0.011	0.717	-0.050	0.072	0.020	0.527	-0.042	0.081	-1.239	0.141	-2.896	0.419
3-hydroxybutyrate	151	0.064	0.024	0.009	0.119	-0.029	0.184	-0.072	0.014	1.001	0.098	-0.187	2.189
**Fluid balance**													
Creatinine	152	-0.019	0.546	-0.082	0.044	-0.004	0.858	-0.044	0.037	-0.296	0.608	-1.436	0.844
Albumin	153	-0.002	0.946	-0.060	0.056	-0.039	0.288	-0.110	0.033	0.743	0.449	-1.195	2.681
**Inflammation markers**													
Glycoprotein acetyls	153	0.116	**0.001**	0.050	0.182	0.000	0.997	-0.041	0.041	0.387	0.490	-0.722	1.496

Used model: Metabolite = $\beta0 + \beta1 * BMI + \beta2 * (SLMxBMI) + \beta3 * SLM + \beta4 * Age + \beta5 * Gender + \beta6 * Status + \beta7 *$ Lipid lowering medication + $\beta8 *$ Antihypertensive medication + $u1 *$ Household + ε
BMI reports $\beta1$, SLM x BMI reports $\beta2$; SLM reports $\beta3$
* Bold p-values indicate significant associations after FDR correction. † % of total fatty acids
SLM, standardized liquid meal; Double bonds/fatty acids, the number of double bonds in fatty acids. VLDL, very low density lipoprotein; IDL, intermediate density lipoprotein; LDL, low density lipoprotein; HDL, high density lipoprotein.

Supplementary Table S5 │Effect size of the association between diastolic blood pressure and metabolites before and after the standardized liquid meal

		fasting				postprandial			
	N	B	p-value*	95% CI		B	p-value*	95% CI	
**Fatty acids**									
Total fatty acids	147	0.028	0.005	0.009	0.048	0.026	0.003	0.009	0.044
Fatty acid chain length	147	-0.002	0.857	-0.019	0.016	0.001	0.948	-0.026	0.028
Double bonds / fatty acids	147	-0.030	**0.001**	-0.047	-0.013	-0.034	0.007	-0.058	-0.009
Docosahexaenoic acid	147	-0.013	0.316	-0.038	0.012	-0.015	0.245	-0.039	0.010
Linoleic acid	147	0.027	0.002	0.010	0.045	0.025	0.003	0.009	0.041
Omega-3 fatty acids	147	-0.002	0.879	-0.026	0.022	-0.004	0.734	-0.026	0.019
Omega-6 fatty acids	147	0.023	0.016	0.004	0.042	0.021	0.014	0.004	0.038
Polyunsaturated fatty acids	147	0.021	0.036	0.001	0.040	0.019	0.036	0.001	0.036
Monounsaturated fatty acids	147	0.029	0.003	0.010	0.048	0.030	0.001	0.013	0.047
Saturated fatty acids	147	0.027	0.005	0.008	0.046	0.023	0.025	0.003	0.043
**Fatty acids, relative to total fatty acids**									
Omega-3 fatty acids (%)†	147	-0.027	0.019	-0.049	-0.005	-0.028	0.023	-0.052	-0.004
Omega-6 fatty acids (%)†	147	-0.013	0.134	-0.031	0.004	-0.013	0.173	-0.032	0.006
Polyunsaturated fatty acids (%)†	147	-0.020	0.021	-0.036	-0.003	-0.020	0.060	-0.040	0.001
Monounsaturated fatty acids (%)†	147	0.019	0.029	0.002	0.037	0.023	0.020	0.004	0.042
Saturated fatty acids (%)†	147	0.002	0.833	-0.015	0.019	-0.006	0.676	-0.037	0.024
**Cholesterol**									
Total cholesterol	154	0.017	0.062	-0.001	0.036	0.014	0.085	-0.002	0.030
non HDL cholesterol	154	0.021	0.019	0.004	0.039	0.017	0.035	0.001	0.032
VLDL cholesterol	154	0.024	0.010	0.006	0.043	0.019	0.016	0.004	0.034
IDL cholesterol	154	0.017	0.057	-0.001	0.034	0.014	0.070	-0.001	0.028
LDL cholesterol	154	0.018	0.042	0.001	0.035	0.013	0.101	-0.003	0.029
HDL cholesterol	154	-0.008	0.421	-0.027	0.011	-0.007	0.478	-0.027	0.013
Cholesterol esterification	147	0.018	0.052	0.000	0.037	0.015	0.060	-0.001	0.031

Supplementary Table S5 (continued)|Effect size of the association between diastolic blood pressure and metabolites before and after the standardized liquid meal

		fasting				postprandial			
	N	B	p-value*	95% CI		B	p-value*	95% CI	
**Lipoprotein lipid concentration**									
Extremely large VLDL	150	0.025	0.008	0.007	0.043	0.034	0.006	0.010	0.057
Very large VLDL	149	0.026	0.010	0.006	0.046	0.030	0.009	0.008	0.052
Large VLDL	151	0.027	0.015	0.005	0.048	0.026	0.009	0.007	0.045
Medium VLDL	154	0.028	0.006	0.008	0.047	0.023	0.005	0.007	0.039
Small VLDL	154	0.024	0.013	0.005	0.043	0.018	0.022	0.003	0.034
Very small VLDL	154	0.018	0.063	-0.001	0.037	0.013	0.082	-0.002	0.028
IDL	154	0.017	0.064	-0.001	0.035	0.014	0.074	-0.001	0.030
Large LDL	154	0.018	0.046	0.000	0.036	0.015	0.073	-0.001	0.031
Medium LDL	154	0.019	0.037	0.001	0.036	0.014	0.089	-0.002	0.031
Small LDL	154	0.018	0.042	0.001	0.036	0.015	0.089	-0.002	0.032
Very large HDL	154	-0.011	0.233	-0.030	0.007	-0.007	0.398	-0.022	0.009
Large HDL	149	-0.007	0.389	-0.025	0.010	-0.006	0.493	-0.023	0.011
Medium HDL	154	0.005	0.632	-0.015	0.024	0.009	0.449	-0.014	0.031
Small HDL	154	0.012	0.205	-0.006	0.030	0.008	0.445	-0.013	0.028
**Lipoprotein particle size**									
VLDL diameter	154	0.026	0.009	0.006	0.045	0.024	0.005	0.007	0.041
LDL diameter	154	-0.007	0.471	-0.025	0.012	-0.004	0.642	-0.023	0.014
HDL diameter	154	-0.011	0.203	-0.028	0.006	-0.008	0.341	-0.025	0.009
**Apolipoproteins and lipids**									
Triglycerides	154	0.027	0.007	0.008	0.046	0.025	0.005	0.008	0.042
Total phosphoglyceride	147	0.018	0.054	0.000	0.037	0.015	0.082	-0.002	0.033
Phosphatidylcholines	147	0.019	0.050	0.000	0.038	0.016	0.073	-0.002	0.034
Sphingomyelins	147	0.006	0.500	-0.012	0.025	0.002	0.850	-0.016	0.019
Apolipoprotein A-I	154	0.002	0.823	-0.016	0.020	0.003	0.741	-0.016	0.022
Apolipoprotein B	154	0.024	0.013	0.005	0.043	0.020	0.015	0.004	0.036

Supplementary Table S5 (continued)|Effect size of the association between diastolic blood pressure and metabolites before and after the standardized liquid meal

		fasting				postprandial			
	N	B	p-value*	95% CI		B	p-value*	95% CI	
**Glycolysis related metabolites**									
Glucose	154	0.011	0.051	0.000	0.022	0.017	0.117	-0.004	0.038
Lactate	154	0.017	0.045	0.000	0.034	0.011	0.342	-0.012	0.035
Pyruvate	152	0.003	0.786	-0.017	0.022	0.015	0.161	-0.006	0.037
Citrate	154	0.020	0.043	0.001	0.039	0.017	0.148	-0.006	0.040
Glycerol	152	0.007	0.149	-0.003	0.017	0.019	0.047	0.000	0.039
**Amino acids**									
Alanine	152	0.006	0.494	-0.011	0.023	0.005	0.594	-0.015	0.025
Glutamine	152	0.008	0.429	-0.012	0.029	0.010	0.348	-0.011	0.030
Glycine	136	-0.017	0.037	-0.033	-0.001	-0.014	0.162	-0.034	0.006
Histidine	154	0.006	0.511	-0.012	0.024	0.002	0.806	-0.017	0.022
Isoleucine	151	0.012	0.238	-0.008	0.031	0.009	0.313	-0.008	0.026
Leucine	153	0.006	0.465	-0.010	0.021	0.007	0.418	-0.010	0.023
Valine	152	0.001	0.954	-0.021	0.022	0.003	0.818	-0.020	0.026
Phenylalanine	154	0.004	0.721	-0.017	0.025	0.001	0.947	-0.022	0.024
Tyrosine	154	0.009	0.393	-0.012	0.030	0.009	0.412	-0.012	0.030
**Ketone bodies**									
Acetate	154	0.020	0.217	-0.012	0.052	0.023	0.069	-0.002	0.048
Acetoacetate	154	0.009	0.369	-0.010	0.028	0.005	0.658	-0.016	0.025
3-hydroxybutyrate	152	0.012	0.243	-0.008	0.032	0.006	0.553	-0.014	0.025
**Fluid balance**									
Creatinine	153	0.008	0.409	-0.011	0.028	0.006	0.584	-0.015	0.027
Albumin	154	0.007	0.460	-0.012	0.026	0.009	0.481	-0.016	0.034
**Inflammation markers**									
Glycoprotein acetyls	154	0.019	0.071	-0.002	0.040	0.022	0.039	0.001	0.043

Effect sizes indicate the association magnitudes in units of 1-SD metabolite concentration per 1-mmHg increment in diastolic blood pressure.

* Bold p-values indicate significant associations after FDR correction. † % of total fatty acids
Double bonds/fatty acids: the number of double bonds in fatty acids. VLDL, very low density lipoprotein; IDL, intermediate density lipoprotein; LDL, low density lipoprotein; HDL, high density lipoprotein.

Supplementary Table S6 | Effect size of the association between insulin and metabolites before and after the standardized liquid meal

		fasting				postprandial			
	N	B	p-value*	95% CI		B	p-value*	95% CI	
**Fatty acids**									
Total fatty acids	148	0.043	0.053	0.000	0.086	0.005	0.106	-0.001	0.012
Fatty acid chain length	148	0.011	0.351	-0.013	0.036	0.007	0.044	0.000	0.013
Double bonds / fatty acids	148	-0.039	0.047	-0.078	-0.001	-0.008	**0.015**	-0.014	-0.002
Docosahexaenoic acid	148	0.014	0.432	-0.021	0.048	0.003	0.407	-0.004	0.009
Linoleic acid	148	0.028	0.105	-0.006	0.062	0.003	0.387	-0.004	0.009
Omega-3 fatty acids	148	0.017	0.355	-0.019	0.052	0.003	0.432	-0.004	0.010
Omega-6 fatty acids	148	0.026	0.155	-0.010	0.062	0.002	0.579	-0.005	0.008
Polyunsaturated fatty acids	148	0.026	0.153	-0.010	0.062	0.002	0.544	-0.005	0.009
Monounsaturated fatty acids	148	0.050	0.036	0.003	0.096	0.006	0.050	0.000	0.012
Saturated fatty acids	148	0.040	0.051	0.000	0.081	0.006	0.071	-0.001	0.012
**Fatty acids, relative to total fatty acids**									
Omega-3 fatty acids (%)[†]	148	-0.017	0.402	-0.058	0.023	-0.001	0.678	-0.008	0.005
Omega-6 fatty acids (%)[†]	148	-0.038	0.028	-0.071	-0.004	-0.008	**0.005**	-0.013	-0.002
Polyunsaturated fatty acids (%)[†]	148	-0.040	0.035	-0.078	-0.003	-0.008	**0.009**	-0.013	-0.002
Monounsaturated fatty acids (%)[†]	148	0.040	0.054	-0.001	0.080	0.005	0.091	-0.001	0.011
Saturated fatty acids (%)[†]	148	-0.001	0.946	-0.026	0.024	0.003	0.397	-0.004	0.009
**Cholesterol**									
Total cholesterol	155	0.008	0.622	-0.024	0.040	0.000	0.918	-0.006	0.007
non HDL cholesterol	155	0.033	0.048	0.000	0.066	0.002	0.502	-0.005	0.009
VLDL cholesterol	155	0.058	**0.004**	0.019	0.096	0.004	0.124	-0.001	0.010
IDL cholesterol	155	0.014	0.393	-0.018	0.045	0.000	0.933	-0.007	0.007
LDL cholesterol	155	0.022	0.163	-0.009	0.052	0.001	0.700	-0.006	0.008
HDL cholesterol	155	-0.070	**0.001**	-0.111	-0.030	-0.007	**0.002**	-0.012	-0.003
Cholesterol esterification	148	0.009	0.571	-0.024	0.043	0.001	0.829	-0.006	0.008

Supplementary Table S6 (continued) | Effect size of the association between insulin and metabolites before and after the standardized liquid meal

		fasting				postprandial			
	N	B	p-value*	95% CI		B	p-value*	95% CI	
**Lipoprotein lipid concentration**									
Extremely large VLDL	151	0.064	**0.014**	0.013	0.114	0.009	**0.008**	0.002	0.016
Very large VLDL	150	0.073	**0.018**	0.013	0.133	0.009	**0.003**	0.003	0.015
Large VLDL	152	0.083	**0.006**	0.025	0.142	0.009	**0.001**	0.004	0.015
Medium VLDL	155	0.081	**1.36E-04**	0.040	0.121	0.008	**0.002**	0.003	0.013
Small VLDL	155	0.065	**0.001**	0.026	0.104	0.006	0.040	0.000	0.011
Very small VLDL	155	0.034	0.051	0.000	0.068	0.002	0.538	-0.004	0.008
IDL	155	0.016	0.324	-0.016	0.049	0.000	0.900	-0.007	0.008
Large LDL	155	0.021	0.210	-0.012	0.053	0.001	0.751	-0.006	0.008
Medium LDL	155	0.027	0.088	-0.004	0.058	0.002	0.572	-0.005	0.009
Small LDL	155	0.026	0.094	-0.005	0.057	0.003	0.448	-0.004	0.010
Very large HDL	155	-0.063	**4.20E-04**	-0.097	-0.029	-0.005	**0.010**	-0.010	-0.001
Large HDL	150	-0.059	**0.001**	-0.094	-0.025	-0.006	**0.014**	-0.011	-0.001
Medium HDL	155	-0.038	**0.020**	-0.069	-0.006	-0.004	0.027	-0.008	-0.001
Small HDL	155	0.012	0.415	-0.017	0.041	0.001	0.635	-0.003	0.005
**Lipoprotein particle size**									
VLDL diameter	155	0.082	**2.33E-04**	0.040	0.125	0.009	**7.45E-05**	0.005	0.014
LDL diameter	155	-0.027	0.046	-0.053	-0.001	-0.006	0.018	-0.010	-0.001
HDL diameter	155	-0.074	**4.78E-05**	-0.109	-0.040	-0.008	**4.23E-04**	-0.012	-0.004
**Apolipoproteins and lipids**									
Triglycerides	155	0.074	**0.001**	0.032	0.116	0.008	**0.003**	0.003	0.014
Total phosphoglyceride	148	-0.004	0.804	-0.038	0.030	-0.001	0.688	-0.006	0.004
Phosphatidylcholines	148	-0.005	0.771	-0.037	0.027	-0.001	0.641	-0.006	0.004
Sphingomyelins	148	0.007	0.696	-0.026	0.039	-0.001	0.770	-0.008	0.006
Apolipoprotein A-I	155	-0.048	**0.004**	-0.080	-0.015	-0.004	0.040	-0.009	0.000
Apolipoprotein B	155	0.049	**0.013**	0.011	0.088	0.004	0.204	-0.002	0.011

Supplementary Table S6 (continued)| Effect size of the association between insulin and metabolites before and after the standardized liquid meal

		fasting				postprandial			
	N	B	p-value*	95% CI		B	p-value*	95% CI	
**Glycolysis related metabolites**									
Glucose	155	0.029	**0.005**	0.009	0.049	0.006	0.049	0.000	0.012
Lactate	155	0.049	**2.46E-05**	0.027	0.072	0.008	**0.007**	0.002	0.014
Pyruvate	153	0.048	**0.005**	0.015	0.080	0.005	0.151	-0.002	0.011
Citrate	155	0.041	**0.016**	0.008	0.073	0.007	**0.003**	0.003	0.012
Glycerol	153	0.028	**0.001**	0.011	0.045	0.006	**0.001**	0.003	0.010
**Amino acids**									
Alanine	153	0.025	0.075	-0.003	0.052	0.007	**0.006**	0.002	0.012
Glutamine	153	-0.022	0.219	-0.057	0.013	-0.004	0.213	-0.011	0.002
Glycine	137	-0.024	0.063	-0.049	0.001	-0.003	0.297	-0.008	0.002
Histidine	155	0.018	0.251	-0.013	0.049	-0.001	0.672	-0.007	0.004
Isoleucine	152	0.079	**6.91E-06**	0.046	0.112	0.009	**2.73E-06**	0.006	0.013
Leucine	154	0.049	**3.38E-05**	0.027	0.071	0.008	**1.87E-05**	0.004	0.012
Valine	153	0.060	**2.41E-06**	0.036	0.084	0.005	0.039	0.000	0.010
Phenylalanine	155	0.065	**9.19E-06**	0.037	0.093	0.009	**0.001**	0.004	0.014
Tyrosine	155	0.060	**4.06E-06**	0.035	0.084	0.009	**3.69E-04**	0.004	0.015
**Ketone bodies**									
Acetate	155	0.008	0.671	-0.031	0.048	-0.001	0.496	-0.005	0.002
Acetoacetate	155	0.013	0.400	-0.018	0.045	-0.004	0.136	-0.009	0.001
3-hydroxybutyrate	153	0.054	**0.001**	0.023	0.086	0.006	**0.008**	0.002	0.010
**Fluid balance**									
Creatinine	154	0.019	0.132	-0.006	0.043	0.001	0.453	-0.002	0.005
Albumin	155	0.011	0.411	-0.016	0.038	-0.001	0.767	-0.008	0.006
**Inflammation markers**									
Glycoprotein acetyls	155	0.088	**2.04E-04**	0.043	0.133	0.007	0.057	0.000	0.015

Effect sizes indicate the association magnitudes in units of 1-SD metabolite concentration per 1-MU/L increment in insulin.
* Bold p-values indicate significant associations after FDR correction. † % of total fatty acids

Double bonds/fatty acids: the number of double bonds in fatty acids. VLDL, very low density lipoprotein; IDL, intermediate density lipoprotein; LDL, low density lipoprotein; HDL, high density lipoprotein.

Supplementary Table S7|Nutritional content Nutridrink

per 100 ml:					
**Energy**	150/625	**kcal/kJ**	**Trace elements**		
			Iron	2.4	**mg**
**Fat** (35 En%)	5.8	**g**	Zinc	1.8	**mg**
Saturated fat	0.6	**g**	Copper	0.27	**mg**
Monounsaturated fat	3.5	**g**	Manganese	0.5	**mg**
Polyunsaturated fat	1.7	**g**	Fluorine	0.15	**mg**
Linoleic acid	1.4	**g**	Molybdenum	15	**µg**
α-Linolenic acid	0.27	**g**	Selenium	8.6	**µg**
Arachidonic acid	-	**mg**	Chromium	10	**µg**
Docosahexaenoic acid	-	**mg**	Iodine	20	**µg**
Eicosapentaenoic acid	-	**mg**			
			**Vitamins**		
**Carbohydrates** (49 En%)	18.4	**g**	A	123	**µg-RE**
Glucose	0.1	**g**	Carotenoids	0.3	**mg**
Fructose	-	**g**	D	1.1	**µg**
Lactose	<0.025	**g**	E	1.9	**mg α-TE**
Maltose	0.6	**g**	K	8	**µg**
Sacharose	6	**g**	Thiamine	0.23	**mg**
Polysacharose	11.3	**g**	Riboflavin	0.24	**mg**
Others	0.4	**g**	Niacin	2.7	**mg NE**
			Pantothenic acid	0.8	**mg**
**Fibers**	-	**g**			
			**Others**		
**Protein** (16 En%)	5.9	**g**	Carnitine	-	**mg**
Casein	5.9	**g**	Choline	55	**mg**
Whey protein	-	**g**	Taurine	-	**mg**
**Salt**	0.23	**g**	**Osmolarity**	455	mOsmol/l
**Liquids**	78	**ml**			
**Minerals**					
Sodium	90	**mg**			
Potassium	159	**mg**			
Chlorine	87	**mg**			
Calcium	91	**mg**			
Phosphorus	78	**mg**			
Magnesium	23	**mg**			

Supplementary Table S8│Nutritional content amino acids Nutridrink

per 100 ml:		
L-Alanine	193	mg
L-Arginine	227	mg
L-Asparagine zuur / L-Asparagine	446	mg
L-Cystine	20.8	mg
L-Glutaminezuur / L-Glutamine	1,394	mg
Glycine	115	mg
L-Histidine	186	mg
L-Isoleucine	338	mg
L-Leucine	606	mg
L-Lysine	545	mg
L-Methionine	183	mg
L-Fenylalanine	323	mg
L-Proline	600	mg
L-Serine	381	mg
L-Threonine	279	mg
L-Tryptofaan	82.2	mg
L-Tyrosine	349	mg
L-Valine	432	mg
L-Methione + L-Cystine	204	mg
L-Phenylalanine + L-Tyrosine	673	mg

References

[1] Würtz. P. *et al.* Metabolic signatures of adiposity in young adults: Mendelian randomization analysis and effects of weight change. PLoS. Med. 11. e1001765 (2014).
